# Vapor Swelling of Polymer Brushes Compared to Nongrafted Films

**DOI:** 10.1021/acs.langmuir.2c01889

**Published:** 2022-11-04

**Authors:** Guido C. Ritsema van Eck, Ellen M. Kiens, Lars B. Veldscholte, Maria Brió Pérez, Sissi de Beer

**Affiliations:** Sustainable Polymer Chemistry Group, Department of Molecules & Materials, MESA+ Institute for Nanotechnology, University of Twente, P.O. Box 217, 7500AE Enschede, The Netherlands

## Abstract

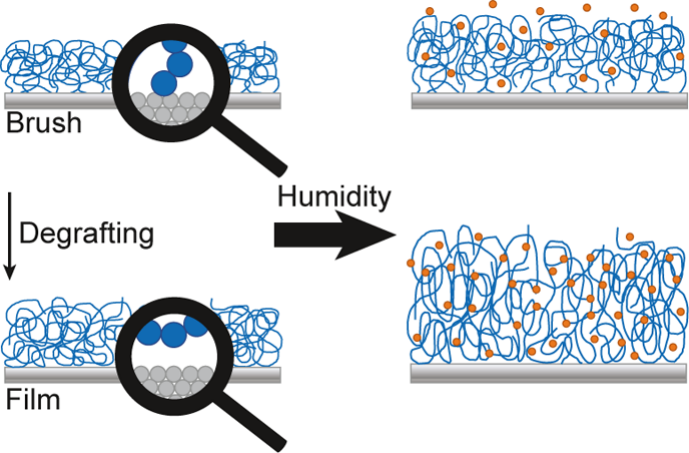

Polymer brushes, coatings of polymers covalently end-grafted to a surface, have been proposed as a more stable alternative to traditional physisorbed coatings. However, when such coatings are applied in settings such as vapor sensing and gas separation technologies, their responsiveness to solvent vapors becomes an important consideration. It can be anticipated that the end-anchoring in polymer brushes reduces the translational entropy of the polymers and instead introduces an entropic penalty against stretching when vapor is absorbed. Therefore, swelling can be expected to be diminished in brushes compared to nongrafted films. Here, we study the effect of the anchoring-constraint on vapor sorption in polymer coatings using coarse-grained molecular dynamics simulations as well as humidity-controlled ellipsometry on chemically identical polymer brushes and nongrafted films. We find a qualitative agreement between simulations and experiments, with both indicating that brushes certainly swell less than physisorbed films, although this effect is minor for common grafting densities. Our results imply that polymer brushes indeed hold great potential for the intended applications.

## Introduction

Polymer coatings can respond to the presence of vapors in air. When a vapor is a good solvent for the polymer, the coatings will absorb the vapor and swell. This property can be utilized for a wide variety of applications, ranging from sensing,^1,2^ where polymer coatings can concentrate the analyte near the sensor surface,^3^ to gas separations,^4,5^ smart moisture management,^6,7^ and lubrication.^8,9^

There are different methods to functionalize substrates with polymers. They can be physisorbed or chemically bound. Although physisorbed, nongrafted films are easy to apply, they exhibit poor adhesive properties due to the low surface energy of most polymer films.^10,11^ This poor adhesion is especially problematic in environments in which the polymer interacts with a liquid or gas and swells. The expansion of the film due to swelling creates stresses such that the coating degrades.^12^ Chemically bound coatings are more stable under swelling. Such coatings can be produced by attaching polymers to the surface by functional groups in their side chains or via their chain ends.

Polymer brushes are a type of chemically bound coating in which polymers are end-grafted to a surface at such densities that the polymer chains stretch away from the substrate to avoid overlapping.^13^ Due to their potential long-term stability,^14−17^ the behavior of these brushes in equilibrium with vapors has recently gained increased attention,^18−23^ and they have been proposed as promising alternatives to physisorbed coatings in gas-based applications. However, the effect of surface grafting on the sorption capabilities of the coating has not been conclusively researched.

**Figure 1:**
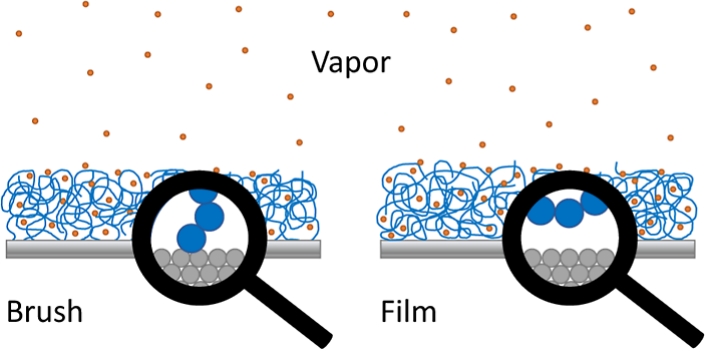
Sketch of the two types of systems that are being studied. Polymer brushes (left) and polymer films (right) are kept in equilibrium with vapor at a constant chemical potential.

In this article, we employ molecular dynamics (MD) simulations and humidity-controlled ellipsometry to address the question of how end-anchoring of polymer chains to a surface affects the swelling properties of the coating. The systems being studied are illustrated in Figure 1. From a theoretical perspective, one can expect that the constraint of end-anchoring the polymers will reduce the vapor sorption of brushes compared to nongrafted films. The end-anchored polymers in a brush incur an entropic penalty when absorbing the vapor, as they must stretch to accommodate the solvent. Thus, they will resist vapor sorption. In contrast, nongrafted chains can rearrange to accommodate solvent in all three dimensions. This leads to a much smaller increase in end-to-end distance, which determines the entropic penalty. Moreover, unbound polymers can gain translational entropy upon absorbing the solvent. Therefore, the expectation is that physisorbed polymer films will swell more than polymer brushes. Yet, it is not clear how significant this effect will be. Experimentally, it has been challenging to study the effect of end-anchoring alone because it is difficult to keep the coating thickness,^24^ molar mass, and dispersity^25^ constant between coatings of different structures. We address this difficulty by hydrolyzing the polymer-surface bonds in some of our polymer brush samples, resulting in nongrafted films of identical molecular weight and dispersity. Additionally, we study vapor sorption using MD simulations, in which all relevant parameters can be set to isolate the effect of surface-grafting the polymers.

In the following, we will first present a theoretical description for vapor solvation of polymer coatings, based on the classical Flory-Huggins model.^26^ Next, we will compare the model to MD simulations of coatings that are exposed to vapors at a constant relative vapor pressure using a grand canonical Monte Carlo (GCMC) procedure. To do so, we build on a simulation procedure recently developed in our group.^27^ Finally, we augment these MD results with experiments in which the swelling of brushes and chemically identical degrafted films are compared.

## Theory

The interaction between solvent and polymers can be described by a mean-field model, based on Flory–Huggins theory of mixing.^26^ This model has been shown to successfully describe the vapor sorption in brushes for one-^27^ and two-component^28^ solvents. In the model, chemical equilibrium between the solvent vapor and the solvent in the polymer layer is assumed, such that a relation between the relative vapor pressure and the solvent volume fraction in the coating can be found. In this section, we will derive two distinct equations for brushes and physisorbed films in contact with solvent vapors and we will discuss the differences. We consider the interactions that are short-ranged relative to the film thickness so that interactions with the substrate do not influence bulk swelling behavior.

In Flory–Huggins theory, a solution is described as a lattice of arbitrary but fixed geometry. In the simplest form of the model, the lattice is fully occupied, and each polymer bead or solvent particle occupies exactly one lattice site. This amounts to assuming a constant density for the polymer solution. Particles are distributed over the lattice randomly, in such a way that particles along a polymer backbone are connected. Using a mean-field assumption for the local composition of the solution, a free energy of mixing can be derived, in which the first two addends represent the combinatorial entropy of mixing and the third represents the enthalpy of mixing relative to the pure bulk solvent and polymer. Here, *n* is the number of molecules, ϕ is the site fraction of the polymer (denoted with subscript p) or solvent (denoted with subscript s), *k*_B_ is the Boltzmann constant, and *T* is the temperature. χ is the well-known Flory–Huggins parameter, which in the ideal theoretical case represents the interchange energy per site between bulk phases of the polymer and the solvent. An additional subscript *f* is appended to all quantities to indicate that this expression describes the nongrafted film, in contrast to the polymer brush (denoted in latter equations by subscript b).
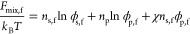
1

The free energy expression described above cannot be used for polymer brushes, since it does not take grafting effects into account. Since chains in a polymer brush are anchored to the surface, they do not possess any translational entropy. The second term in eq 1, which results from the translational freedom gained by the polymer upon solvation, should therefore be eliminated. Moreover, an additional term to include the entropic penalty of stretching of the polymer chains perpendicular to the surface must be added.^29^ This results in a free energy expression for the brush
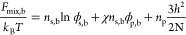
2where *N* is the degree of polymerization and *h* is the brush height, expressed in monomer lengths. The last term in this expression can be rewritten by assuming a uniform brush density. This means that the height of the brush is proportional to the total number of particles per unit area
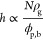
3where ρ_g_ is the grafting density, the number of chains per unit area. Substituting this expression for *h* in the free energy expression gives
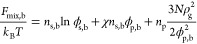
4Due to this additional force opposing solvent uptake, we expect polymer brushes to absorb less solvent vapor than nongrafted films. This difference should be most pronounced at high solvent uptake (i.e., low ϕ_p,b_), where the stretching term rapidly increases.

We obtain predicted sorption isotherms for both the brush and the nongrafted film by taking the derivatives of the free energy expressions with respect to *n*_s_, which amounts to the chemical potential for solvent in the system. Assuming the solvent vapor outside the coating to be an ideal gas, for which the chemical potential is given by
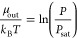
5we obtain equilibrium conditions by equating the chemical potential of solvent inside and outside the coating. The resulting relations between the vapor pressure and solvent uptake are
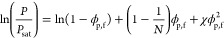
6for the nongrafted film and
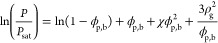
7for the polymer brush. While real gases typically deviate from ideality at high concentrations, this only influences the chemical potential of vapor outside the coating and so comparisons between brushes and films at any given pressure should remain valid.

**Figure 2:**
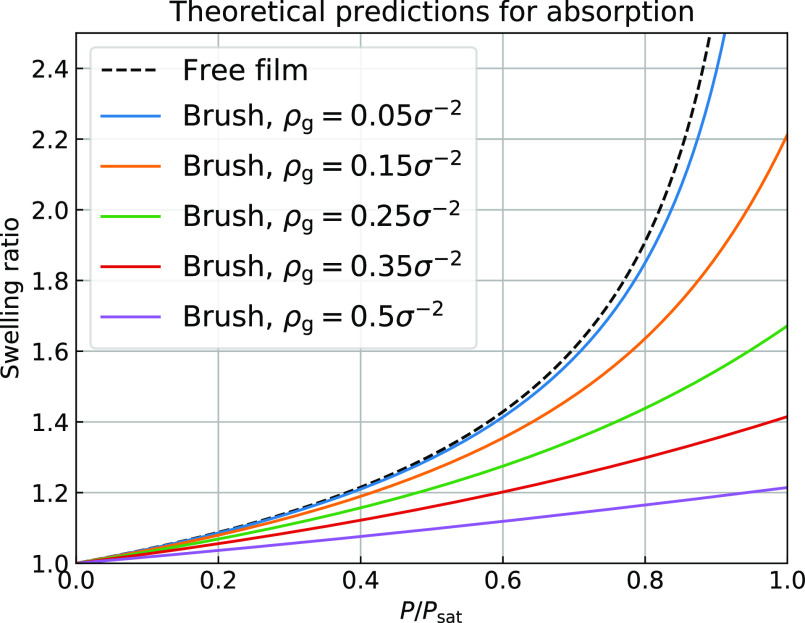
Theoretical predictions for the swelling of the coatings as a function of vapor pressure according to Flory–Huggins theory for film and brush with different grafting densities and χ = 0 and *N* = 100.

The expressions derived above give the shape of the sorption isotherm for any fixed value of χ. We may also relate the solvent fraction to a swelling ratio, which is more experimentally accessible, via the relation: 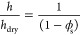
8This relation applies for any definition of the brush height that scales linearly with the total mass per unit area. Figure 2 displays the swelling ratio of a nongrafted film and polymer brushes of various grafting densities in an athermal solvent, as predicted by this model. These isotherms show reduced sorption in polymer brushes relative to nongrafted films. Since the entropic penalty of stretching the polymer chains increases with the grafting density, solvent absorption is expected to decrease with the grafting density. To test if brushes indeed absorb less solvent than films, we have set up MD simulations as explained in the next section.

## Model and Methods

### Simulations

We investigate the sorption behavior of polymer brushes and films using an alternating MD and GCMC procedure, previously described in ref (27). In this combined procedure, the MD simulations model the evolution of the polymer–vapor system. Periodic GCMC sweeps maintain a constant chemical potential of the solvent vapor in a region above the brush. All simulations are performed using the LAMMPS package.^30^

We describe our system in the system of reduced units derived from the Lennard-Jones (LJ) potential. Units of length (σ) and energy (ϵ) are derived from the zero-crossing distance and potential well depth of a reference LJ potential. A detailed discussion of the potentials used in our simulations can be found in the Supporting Information.

Our simulations consist of a box of 30 σ × 30 σ × 111 σ in *x*, *y*, *z* with periodic boundary conditions in the *x* and *y* dimensions. This box is closed off at the top in *z* by a mathematical wall, which imposes a strong (100 ϵσ^–2^) harmonic repulsion on particles within 1 σ of the box edge. At the bottom of the box in *z*, the system is similarly bound by a 9–3 LJ potential, which effectively models a perfectly flat, homogeneous wall of LJ particles. The potential well depth ϵ_93_ and the zero-crossing distance σ are set to 1, and the potential is cut off at a distance of 2.5 σ.

Simulated coatings are set up in a similar manner, so results are maximally comparable between brushes and nongrafted films. 135 polymer chains with chain length *N* = 100 are placed above the wall, amounting to an areal density of 0.15 chain σ^–2^. This density ensures that the mean gyration radius of a polymer chain in the globule state is larger than the mean distance between polymers, meaning that chains in the grafted system will always experience excluded volume interactions. These polymer chains are represented by a freely joined bead-spring model, based on the coarse-grained model introduced by Kremer and Grest.^31^ All interparticle interactions are truncated at 2.5σ, resulting in attractive interactions from the potential minimum up to the cutoff distance. This results in a poor implicit medium, representing the fact that air is an unfavorable medium for polymers. We assume that the LJ potentials we use represent arbitrary short-ranged interactions. Hence, we do not account for combining rules in our parameter selection. For most interactions, we simply use ϵ = 1. However, we vary the polymer–solvent interaction strength ϵ_ps_ over a range from 0.7 to 1.4 (at constant ϵ_pp_ = 1), and the polymer self-interaction ϵ_pp_ from 0.8 to 1.2 (at constant ϵ_ps_ = 1).

A GCMC region of 40 σ in *z*, spanning the whole width of the box, is defined near the top of the box. This region exchanges vapor particles with a virtual atmosphere through the aforementioned GCMC procedure, in which particle insertions and deletions are evaluated according to a Metropolis criterion. GCMC sweeps are performed every 10000 timesteps, with 1000 attempted insertions and deletions per sweep.

This system is initially set up with the polymer chains in a fully extended configuration. In this initial state, each chain is attached at one end to an extra, “frozen” particle near the wall through a finitely extensible nonlinearly elastic (FENE) bond. While the resulting configuration is brush-like, it also prevents the unequilibrated polymers from detaching from the surface in the free film case. This system is equilibrated first by energy minimization through the conjugate gradient method. This is followed by 10,000 timesteps of dynamics, during which a maximum particle velocity of 1 σ per timestep is imposed. The system is thermostatted by a Langevin thermostat with a damping parameter of 1000 τ (LJ-derived time units) during this run. Afterward, a second minimization and 5,00,000 more timesteps of dynamics are performed. During this second run, the damping parameter of the thermostat is set to 100 τ and no velocity limit is applied. In this way, we relax the polymer chains from their initial, extended state to a more entropically realistic one while ensuring that they remain at the surface. At this point, we change the thermostat to a chain of three Nosé–Hoover thermostats (which accurately samples the canonical ensemble^32^) and integrate the system for another 1 million timesteps at an LJ temperature of 0.85. This temperature has been previously verified to allow vapor–liquid coexistence for our vapor parameters.^33^ We use the resulting system as the initial state for our polymer brush simulations. The initial configuration for the polymer film simulations is produced by deleting the previously introduced frozen particles and allowing the system to re-equilibrate for another 1 million timesteps. Next, we perform production runs of 20 million timesteps with the aforementioned GCMC procedure, using the same thermostat. Both the equilibration and production runs use a two-level rRespa integrator,^34^ with an outer timestep of 0.015 τ and an inner timestep length of 0.0075 τ. Nonbonded pair interactions are computed in the outer timestep, while bonded interactions are computed in the inner timestep. These simulations are performed separately for all different combinations of ϵ_pp_ and ϵ_ps_ values. Additionally, sorption isotherms are obtained for ϵ_pp_ = 0.9 and ϵ_ps_ = 1.0, 1.4 by changing the chemical potential of the virtual reservoir in the GCMC procedure (and hence the relative solvent pressure *P*/*P*_sat_).

For all runs, density profiles of monomer and solvent particles are collected over the last 4 million timesteps of the simulation, to ensure an equilibrated solvent distribution. We verify that systems are equilibrated by ensuring that the density profiles no longer meaningfully change between the beginning and end of this collection period. In these profiles, we define the brush height as the inflection point of the brush density profile. Absorption is quantified by integrating the solvent concentration from 5 σ above the grafting plane up to the brush height, to exclude possible effects of the mathematical wall. Adsorption is defined by integrating the solvent concentration from the brush height up to the boundary of the solvent layer. We define this boundary as the point where the gradient of the solvent density reaches 0.002. This value is empirically determined to exclude fluctuations in the vapor bulk while including almost all condensed solvent in the adsorption layer.

### Experiments

#### Materials

Copper(I) bromide (CuBr, Merck, ≥ 98%) is purified in glacial acetic acid by continuous stirring until the suspension solution is pale white. After that, the acetic acid is removed, followed by multiple washing cycles with ethanol. Next, the resulting powder is dried in a vacuum oven (room temperature, overnight). 3-Sulfopropyl methacrylate potassium salt (SPMAK, 98%), (3-aminopropyl)triethoxysilane (APTES, 98%), α-bromoisobutyryl bromide (BiBB, 98%), 2,2′-bipyridyl (≥98%), triethylamine (TEA, ≥ 98%), and ethyl α-bromoisobutyrate (EBiB, ≥ 98%) are purchased from Merck and used as received. Methanol (ACS reagent) and toluene (ACS reagent) are purchased from Biosolve and used as received. MilliQ water purified from a MilliQ Advantage A10 purification system (Millipore, Billerica, MA, USA) is used.

#### Synthesis of Silane-Anchored poly(SPMA) Brushes

The followed synthetic route for the grafting of poly(SPMA) brushes from silicon substrates is explained in detail in ref (35). Briefly, (3-aminopropyl)triethoxysilane is deposited by means of chemical vapor deposition on piranha-cleaned substrates, followed by the grafting of α-bromoisobutyryl bromide initiators. Afterward, poly(SPMA) brushes are synthesized by means of surface-initiated atom transfer radical polymerization (SI-ATRP).

To obtain an estimate of the grafting density of our brushes, we perform parallel experiments in which we simultaneously grow brushes in solution and from surfaces by the addition of a sacrificial initiator, ethyl α-bromoisobutyrate (EBiB). Based on the monomer conversion, measured by ^1^H NMR, and the initiator concentration in solution, we obtain an estimated molecular weight of 46.6 kDa for brushes around 15 nm in thickness. This corresponds to an approximate chain density of 0.15 nm^–2^. Although polymerization in solution and from the surface may produce differences in chain length^36^ and polydispersity,^37^ we take this as an order-of-magnitude indication that the surface grafting is sufficiently dense to form a brush. This is also supported by atomic force microscopy (AFM) imaging (Figure S7), where the high chain density and overall layer uniformity can be visualized.

#### Films by Degrafting Brushes

Nongrafted films that are maximally comparable (in terms of thickness, molecular weight, and molecular weight distribution) to the brushes are produced by taking brushes and exposing them to saturated water vapor for an extended period. This reliably degrafts the brushes without dissolving and removing the polymer from the substrate.^22^

The brush samples are stored in an air-tight glass container containing a layer of liquid water for at least 8 weeks, without allowing them to come in contact with the liquid water, after which they are used as is. Degrafting of the brushes is verified by AFM imaging of additional samples (not used in subsequent swelling experiments) in their initial state and after degrafting and rinsing with water and ethanol; after the degrafting and rinsing procedures, only sporadic, thin patches of polymeric material remained, indicating that a large majority of polymer chains had in fact been degrafted and subsequently rinsed off. Considering its low surface coverage and thickness relative to the pristine coatings, we are confident that the remaining fraction of polymer could not cause brush-like behavior in the free coatings. AFM images and height profiles are shown in Supporting Information, Figures S7 and S8.

#### Humidity-Controlled Ellipsometry

The humidity-dependent swelling response of the samples is characterized using a J.A. Woollam M-2000X spectroscopic ellipsometer with a 5 mL heated liquid cell (J.A. Woollam) connected to an OpenHumidistat^38^ humidity controller. The cell’s heating feature is not used.

Closed-loop control over the humidity of an air stream is provided by the humidistat. Since it is not feasible to fit a humidity sensor (for feedback) in the ellipsometer’s liquid cell, it is used with an universal prechamber containing the humidity sensor. The outlet of this prechamber is connected to the liquid cell of the ellipsometer.

Ellipsometry measurements are performed at wavelengths between 350 and 1000 nm, at an angle of incidence of 75°, in in situ mode, which acquires data continuously over time. At the same time, the humidity setpoint on the humidistat is scanned in steps of 10 percent-point from 10 to 60% and in steps of 5 percent-point from 60 to 90%. This procedure is chosen for better resolution and equilibration at higher humidity values because the swelling response of films and brushes is highly superlinear. Every humidity setpoint is held stable for 100 s. Since degrafting of poly(SPMA) brushes in high humidity occurs on a timescale of days,^22^ we do not expect substantial degrafting during these measurements.

The ellipsometric data are fitted to a model composed of a Si substrate, a 1 nm native oxide layer, and a Cauchy layer with (uniaxial) optical anisotropy. The thickness and Cauchy *A*_*xy*_, *A*_*z*_, *B*_*xy*_, and *B*_*z*_ coefficients are fitted. Higher-order coefficients are not used, and our samples are assumed to be optically transparent over the measured wavelength range. Thickness nonuniformity (slight variation of the layer thickness within the measurement spot) is included in the model, and the amount is fitted. Though ellipsometry measurements of brushes in liquid can benefit from explicitly incorporating density gradients over the height of the layer using a graded model,^39,40^ we have shown in an earlier publication that the quality of the fit does not improve enough to justify complicating the fitting model in this way for vapor-solvated brush systems.^23^

The resulting thickness-over-time data are related to the humidity-over-time data from the humidistat. The two independently measured time series are aligned in time using cross-correlation to determine the time delay and interpolated at common time points. Next, the data is filtered to where the humidity is stable, and for each group of thickness-over-time for constant humidity an exponential function (eq 9) is fitted to extract the asymptote (*h*_eq_) using the time constant τ as a fitting parameter. This way, the equilibrium thickness can be estimated even when swelling has not been able to reach full equilibrium within the allowed time.
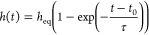
9

The aforementioned procedure is performed for each in situ ellipsometry measurement set. In total, four brush and four film samples are measured in duplicate. The resulting thicknesses are converted to swelling ratios (by dividing by the dry height), and all data for the brushes and films are combined to yield aggregated average swelling ratios as functions of humidity and corresponding confidence intervals for brushes and for films.

## Results and Discussion

In this section, we first discuss the simulation results, starting with density profiles over a range of relative vapor pressures for grafted and nongrafted coatings at fixed interaction energies. Next, we present the swelling ratio as a function of solvent pressure for both brushes and nongrafted films for two different values of the polymer–solvent interaction strength. Finally, we will discuss the experimental results, where we obtained humidity-dependent swelling ratios for brushes and films.

### Simulated Vapor Swelling

**Figure 3:**
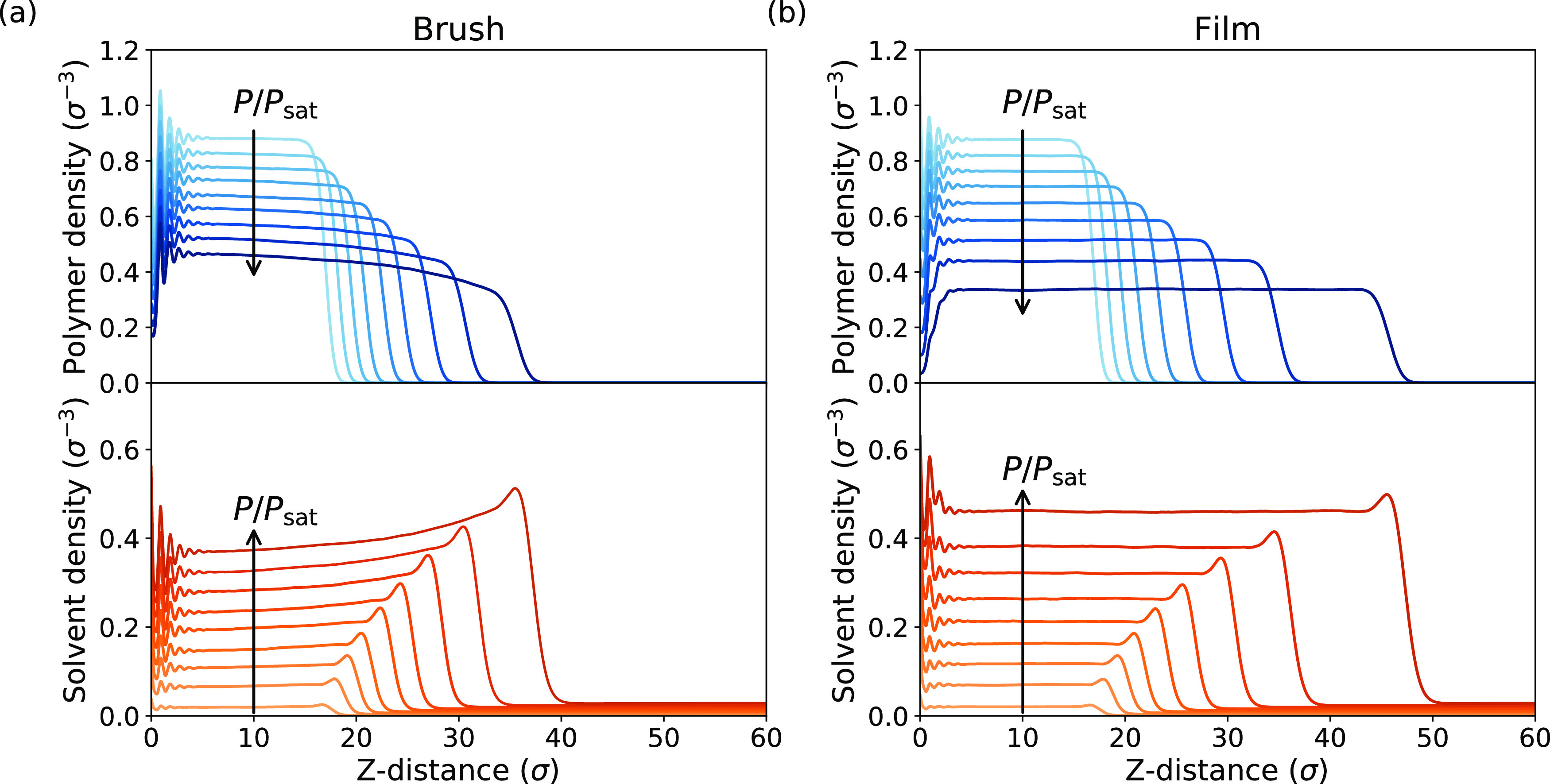
Density profiles of polymer (blue) and solvent (orange) of the brush (a) and film (b) for different vapor pressures and ϵ_ps_ = 1.0, ϵ_pp_ = 0.9. From light to dark, the vapor pressures associated with the lines in the graphs are *P*/*P*_sat_ = 4.9, 15.7, 26.1, 36.4, 48.4, 60.3, 72.7, 85.6, 99.1%.

The density profiles of polymer and solvent as a function of relative vapor pressure are shown in Figure 3. These density profiles are obtained at interaction parameters ϵ_ps_ = 1.0 and ϵ_pp_ = 0.9. In all systems, slight density oscillations appear near the grafting surface, reflecting the formation of layers in the fluid near the wall. Although this layering is amplified by the perfectly flat mathematical wall, it is not unphysical^41^ and has in fact been observed experimentally.^42^ The shape of the density profiles differs significantly between the free film and the brush, particularly at higher solvent pressures. Under these circumstances, the density profile of the brush resembles the parabolic one predicted by classical scaling theories,^13,43^ although neutron reflectometry studies show that the density of vapor-solvated brushes decays more steeply at the outer edge of the brush.^21^ The parabolic profile manifests only at high vapor pressures, since it requires the brush to be highly solvated. Additionally, the polymer–solvent interactions in these systems are highly favorable, translating to negative values of χ. These strong interactions may reduce the impact of entropic contributions on the profile shape, compared to more moderate interaction strengths. The free film, on the other hand, retains a single bulk composition in the entire layer at any given *P*/*P*_sat_. At the highest *P*/*P*_sat_ values of 72.7, 85.6, and 99.1%, the film swells significantly more than the brush.

**Figure 4:**
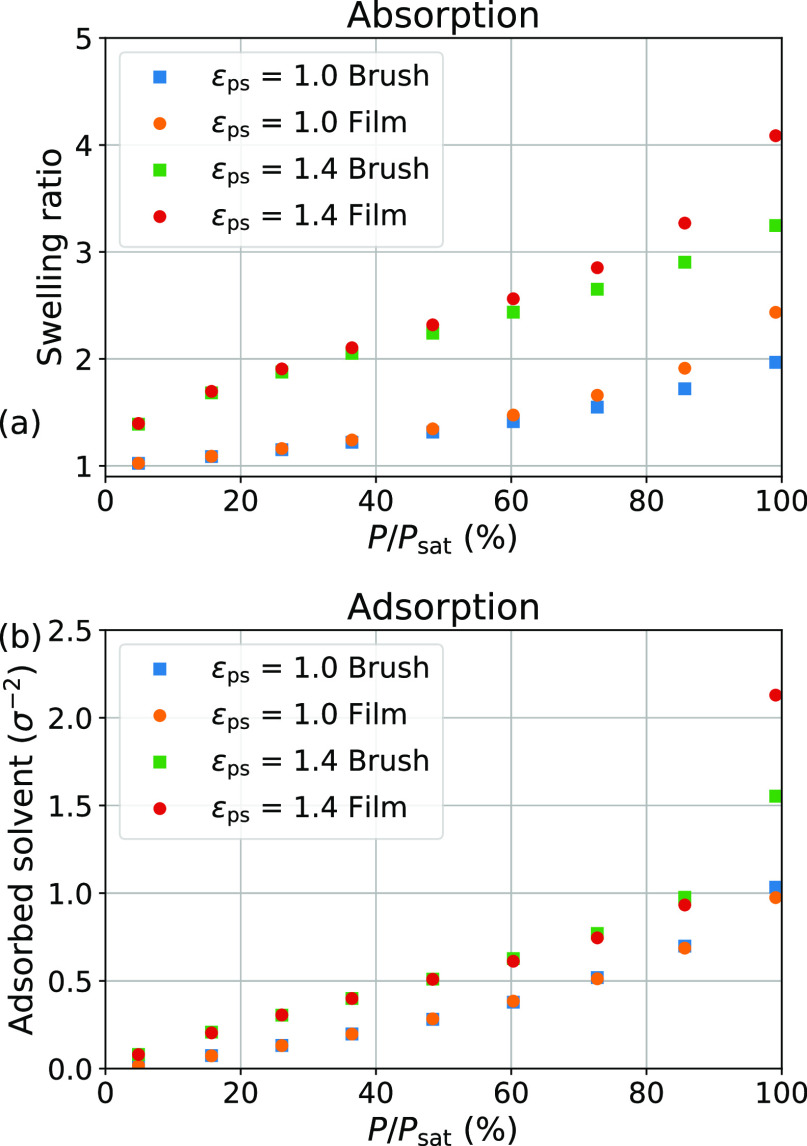
Swelling ratios (a) and amount of adsorbed solvent (b) at varying solvent pressure. Results are presented for the brush and film at ϵ_pp_ = 0.9 and different ϵ_ps_ values.

Brush swelling ratios for ϵ_pp_ = 0.9 and ϵ_ps_ = 1.0, 1.4 are shown as a function of *P*/*P*_sat_ in Figure 4a. Although excluded volume parameters more rigorously describe solvation behavior,^44^ the pairwise interaction energies can be considered equivalent assuming the density of the solution does not change drastically.^45^ These swelling ratios are obtained from solvent and polymer fractions in the brush using eq 8, for easy comparison to the Flory–Huggins model. Since the total density of the coatings varies only slightly over the studied range of interaction parameters and humidities (see Supporting Information, Figures S1–S5), we expect this to be an accurate indication of brush swelling.

We once again observe that the swelling ratios for the brush and the nongrafted system diverge at higher solvent pressures. For the brush, we find a convex swelling curve at ϵ_ps_ = 1.0, shifting to a concave shape at ϵ_ps_ = 1.4. This qualitative change can be explained as a shift from sorption driven by the entropic gain of the solvent entering the polymer layer to a rapidly saturating maximization of polymer–solvent contacts, driven by enthalpy.^33^ The nongrafted film behaves very similarly to the polymer brush at low-solvent pressures and swells slightly more than the brush at high *P*/*P*_sat_ values.

In the limit of vapor saturation, we would expect the condensation of a macroscopic solvent layer, turning the nongrafted film into a dilute polymer solution (cf. Figure 2). However, our model overestimates the sorption at high *P*/*P*_sat_ significantly, and we find strong but finite swelling even for near-saturated vapors. Since Flory–Huggins theory considers only bulk solutions, it seems likely that this overestimation is due to some interfacial effect. For instance, the polymer chains possess less translational entropy than the solvent particles. This also means they may lose less entropy in the presence of the wall, which would favor finite swelling. Alternatively, the discrepancy may have dynamic origins. Even if the dissolution of the polymer film is thermodynamically favorable, entanglements between chains could plausibly prevent polymers from leaving the layer and slow this process down beyond the timescale of our simulations. In addition to absorption, we observe adsorption of solvent onto the surface of the polymer layer. The amount of solvent per unit area outside the polymer bulk, indicative of adsorption, is shown in Figure 4b. The amount of adsorbed solvent increases with the solvent pressure in all cases and does not differ strongly between the brush and the nongrafted film at low pressures. Near saturation, however, more solvent appears to adsorb onto the brush, especially in the ϵ_ps_ = 1.4 case. Whether adsorption occurs is mainly defined by the difference in self-affinity (and by extension surface tension) between the polymer and the solvent. The amount of solvent adsorbed is also influenced by attractive polymer–solvent interactions, however.^27^ This may explain the difference in adsorption between polymer brushes and nongrafted films: since the brush contains less solvent than the nongrafted film, a higher concentration of polymer is available at the brush surface. Since the polymer–solvent interaction is stronger than the solvent self-affinity, the higher polymer concentration favors adsorption of solvent onto the brush surface.

### Humidity-Dependent Swelling of Poly(SPMA)

**Figure 5:**
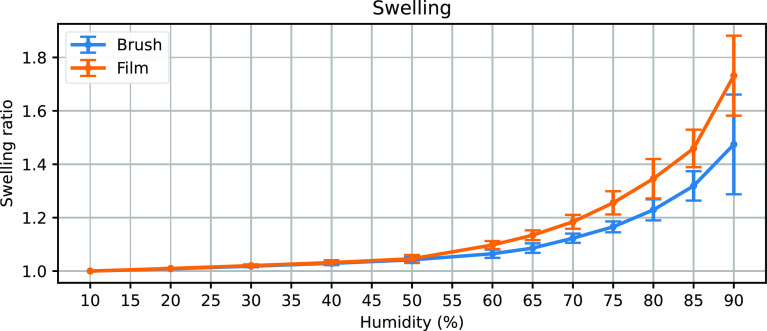
Average swelling behavior of brushes compared to films as measured by humidity-controlled ellipsometry. Lines connecting markers are meant to guide the eye. Error bars denote 95% confidence intervals.

In Figure 5, the swelling of poly(SPMA) brushes and films as a function of humidity, as measured by ellipsometry, is presented. These swelling curves represent aggregated average results for four polymer brush samples and four nongrafted film samples. Both films and brushes display limited swelling at low humidities, and the swelling curves are virtually identical up to 50% relative humidity. At higher humidities, both swelling plots display a concave-upward shape, which is commonly observed for polymer swelling experiments in moderately favorable solvent vapors.^18,19,25,46^ Although factors such as polydispersity could cause deviations from the idealized brushes studied in our simulations,^47,48^ the measured isotherms agree approximately with the simulated ϵ_ps_ = 1.0 case shown in Figure 4a. A significant difference in swelling between the nongrafted films and brushes appears at higher humidity values, with films displaying more relative swelling. This finding also agrees with our theoretical and simulation results. At the highest measured humidity value of 90%, the films swell ∼1.7 × on average, while the average swelling ratio of brushes does not exceed ∼1.5 at that humidity.

The finding that polymer brushes display reduced swelling appears to contradict previous experimental results. McCaig et al. studied the responsiveness to organic vapors of gold-coated silicon nitride nanocantilevers functionalized with drop-cast poly(methyl methacrylate) (PMMA) and PMMA brushes.^24^ When exposed to polar solvent vapors, brush-coated cantilevers displayed significantly increased frequency shifts compared to bare sensors and cantilevers with drop-cast films. However, the authors themselves point out that neither the mass uptake nor the swelling of the polymer film directly correlates with the sensor response. Moreover, synthesis procedures and film heights for the drop-cast films and the polymer brushes differed significantly. Similarly, Galvin and Genzer report higher swelling factors and correspondingly lower χ parameters for brushes in a spectrometric ellipsometry study of poly(2-(dimethylamino)ethyl methacrylate) (PDMAEMA) and PDMAEMA-derived films.^25^ However, this study explicitly does not control the chain length or polydispersity. Galvin and Genzer also pointed out the possibility that the orientation of chains in the polymer brush, which is predominantly normal to the grafting surface, facilitates the formation of diffusion channels for vapor to enter the brush. Finally, they note that their experiments were carried out near the glass-transition temperature of bulk PDMAEMA, which further complicates the interpretation of the results. For both of these studies, it is clear that a direct comparison of swelling in brushes and films is simply outside the scope of the work. Hence, we do not think our findings truly conflict with these previous results.

We also note that the optical anisotropy in brushes behaves differently from that in films. We found that films tend to become optically more isotropic with swelling, while brushes do not. This matches our expectations: the optical anisotropy is related to the preferred alignment of chains.^49^ Chains in films may be “frozen” in an anisotropic state after fast drying processes but become more mobile when solvated by water vapor. In contrast, the grafting of chains in the brushes precludes isotropic orientation even when solvated.

## Conclusions

In this work, we have investigated and compared the swelling behavior of grafted and nongrafted polymer films in (water) vapor, incorporating theory, MD simulations, and experiments. Nongrafted films in these experiments were prepared by degrafting of polymer brushes, ensuring good comparability between the two. Simulation results and experiments both indicate that a polymer brush swells less than the equivalent nongrafted film at all relative humidities, as a result of the constraints imposed by surface-anchoring. We relate these results to a modified Flory–Huggins model, which includes an entropic penalty for stretching of the grafted polymer. This model adequately describes the absorption isotherms obtained from MD simulations, and qualitatively matches experimental results. However, the model overestimates the difference in swelling between polymer brushes and nongrafted films at high humidity. These results further support the potential of polymer brushes for sensing and separation technologies in the gas phase.

## Data Availability

Data underlying this study are openly available through the Open Science Framework, at doi.org/10.17605/OSF·IO/E89Q3.

## Supporting Information Available

Plots of total particle density against *z* for various simulation conditions, full description of the potentials used in MD simulations, discussion on the relation between simulation parameters and the Flory–Huggins parameter, AFM images and height profiles indicating degrafting of the brushes (PDF), and plots of the thickness nonuniformity parameter used in fitting ellipsometry data (PDF)
