# Supplementary material for: Vapor Swelling of Polymer Brushes Compared to Nongrafted Films
Source: Langmuir. 2022 Nov 4;38(45):13763–70. doi: 10.1021/acs.langmuir.2c01889 (PMC9671043; doi:10.1021/acs.langmuir.2c01889)
Supplement: Supplementary file 1 — la2c01889_si_001.pdf [file la2c01889_si_001.pdf]

Supporting information: Vapor Swelling of Polymer Brushes Compared to Non-Grafted Films

Guido C. Ritsema van Eck,[†,‡] Ellen M. Kiens,[†,‡] Lars B. Veldscholte,[†] Maria Brió Pérez,[†] and Sissi de Beer[*,†]

†*Sustainable Polymer Chemistry Group, Department of Molecules & Materials, MESA+ Institute for Nanotechnology, University of Twente, P.O. Box 217, 7500 AE Enschede, The Netherlands*

‡*These authors contributed equally*

E-mail: s.j.a.debeer@utwente.nl

Phone: +31 (0)53 489 3170

Total particle density (as a function of interaction parameters)

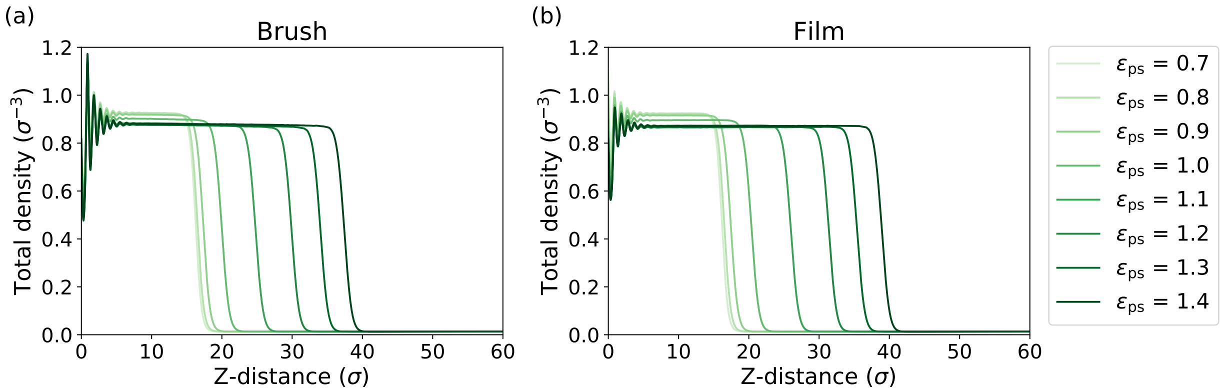

Figure S1: Profiles of the total density for ϵ_{ps} ranging from 0.7 to 1.4 and $\epsilon_{\mathrm{pp}} = 1.0$, $P/P_{\mathrm{sat}} = 50\%$.

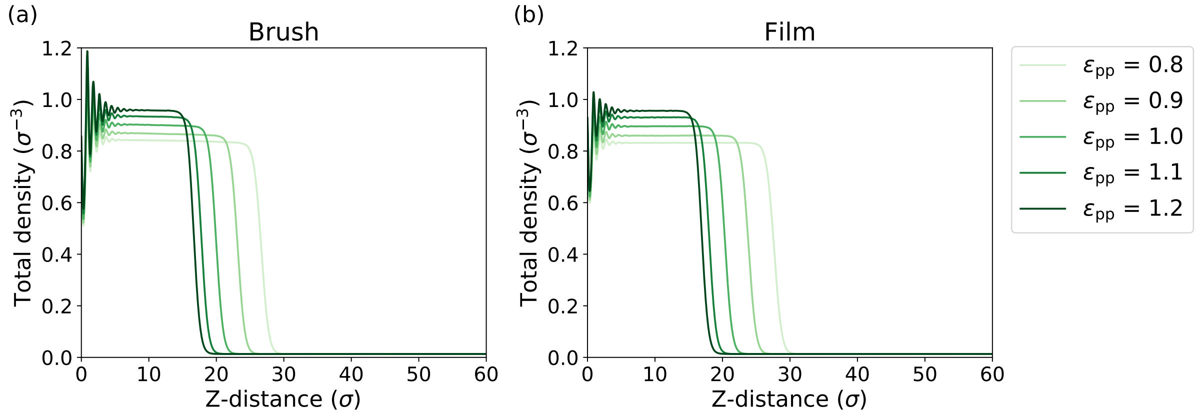

Figure S2: Profiles of the total density for ϵ_{pp} ranging from 0.8 to 1.2 and $\epsilon_{\mathrm{ps}} = 1.0$, $P/P_{\mathrm{sat}} = 50\%$.

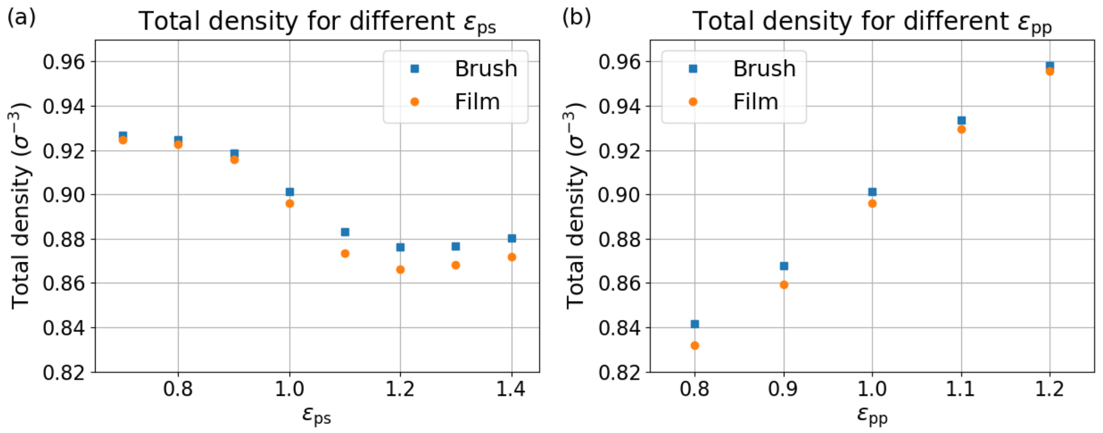

Figure S3: Total density at $z = 10\sigma$ for $P/P_{\mathrm{sat}} = 50\%$ under variation of the polymer-solvent and polymer self- interaction energies.

Total density as a function of P/P_{sat}

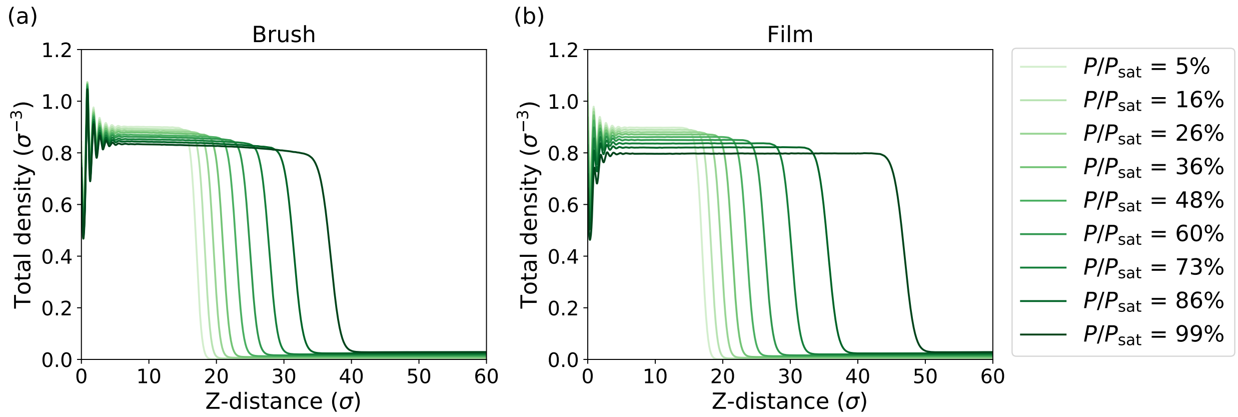

Figure S4: Profiles of the total particle density at different values of solvent activity for $\epsilon_{ps} = 1.0$, $\epsilon_{pp} = 0.9$.

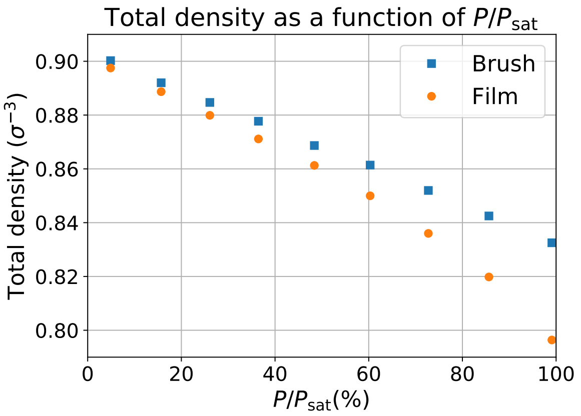

Figure S5: Total density at $z = 10\sigma$ vs P

Relation between simulation parameters and χ

From simulation results, we can obtain an effective χ value by rearranging equations 6 and 7 of the main work as:

$$\chi_\mathrm{b} = -\frac{1}{\phi_\mathrm{p,b}^2}\left(\ln(1-\phi_\mathrm{p,b}) + \phi_\mathrm{p,b} + \frac{3\sigma^2}{\phi_\mathrm{p,b}} - \ln(\frac{P}{P_\mathrm{sat}})\right) \tag{S1}$$

for the brush, and

$$\chi_\mathrm{l} = -\frac{1}{\phi_\mathrm{p,l}^2}\left(\ln(1-\phi_\mathrm{p,l}) + (1-\frac{1}{N})\phi_\mathrm{p,l} - \ln(\frac{P}{P_\mathrm{sat}})\right) \tag{S2}$$

for the non-grafted film.

From a theoretical perspective, we can translate the interaction parameters $\epsilon_\mathrm{pp}, \epsilon_\mathrm{ps}$ and ϵ_ss to a pairwise interchange energy

$$W = -\epsilon_\mathrm{ps} + \frac{1}{2}\left(\epsilon_\mathrm{pp} + \epsilon_\mathrm{ss}\right). \tag{S3}$$

This quantity is related to χ by

$$\chi = \frac{zW}{k_\mathrm{B}T}, \tag{S4}$$

where z is the coordination number for particles in the solution. For constant density of the solution, this implies a linear relation between χ and W. Figure 6 displays χ values recovered from simulations at $P/P_\mathrm{sat} = 50\%$ as well as the best linear fit through the origin. While the simulation results do display the expected linear trend, the obtained χ values are consistently higher than expected. This further indicates that the Flory-Huggins description performs adequately, but overestimates polymer swelling in these systems.

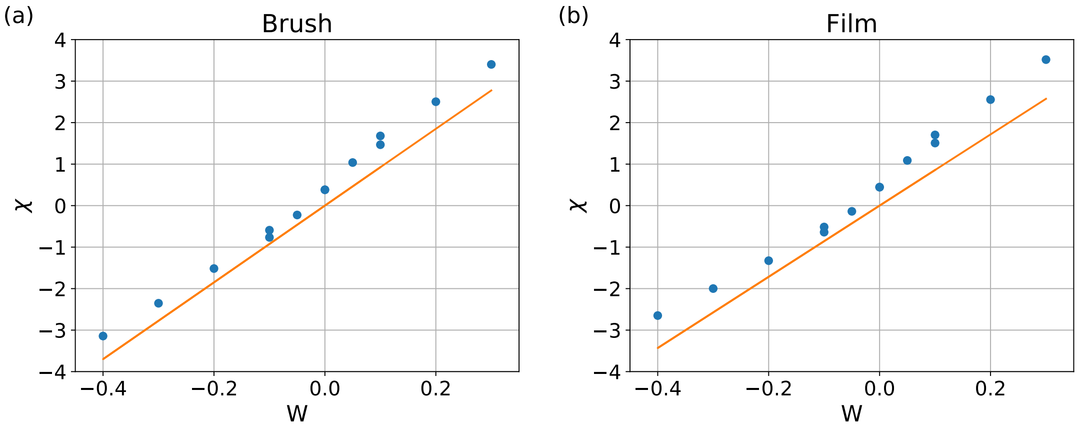

Figure S6: Effective values of the Flory-Huggins parameter as a function of the interchange energy W defined by simulation parameters, for (a) our polymer brush simulations and (b) our non-grafted film simulations.

Details of simulation potentials

Our simulations model polymeric behaviour through the Kremer-Grest bead-spring model,[1] a set of interaction potentials that reproduce typical polymer dynamics and prevent certain unphysical behaviors such as bond-crossing. Non-bonded particles in the system interact through the well-known Lennard-Jones potential. The LJ potential is defined as

$$U_{\mathrm{LJ}}(r) = 4\epsilon \left(\left(\frac{\sigma}{r}\right)^{12} - \left(\frac{\sigma}{r}\right)^{6} \right), \tag{S5}$$

with r being the distance between a given particle pair, ϵ the value of the potential in its minimum, and σ the distance at which the potential crosses zero. This form includes an asymptotically decreasing long-range component, however. To maintain a computationally tractable system, we utilize the truncated and potential-shifted (SP) form:

$$U_{\mathrm{LJ,SP}}(r) = \begin{cases} U_{\mathrm{LJ}}(r) - U_{\mathrm{LJ}}(r_{\mathrm{c}}) & \text{for } r \leq r_{\mathrm{c}} \\ 0 & \text{for } r > r_{\mathrm{c}} \end{cases}, \tag{S6}$$

where r_{c} is the cutoff distance for the interaction.

Particles along a polymer backbone are connected by the combination of a Weeks-Chandler-Anderson (WCA) potential (equivalent to an LJ potential cut off in the minimum at $2^{1/6}\sigma$) and a finitely extensible non-linear elastic (FENE) potential. Hence, the total form of the bonded potential is

$$U_{\mathrm{FENE}}(r) = -0.5KR_0^2 \ln\left(1 - \left(\frac{r}{R_0}\right)^2\right) \tag{S7}$$

$$U_{\mathrm{WCA}}(r) = \begin{cases} U_{\mathrm{LJ}}(r) + \epsilon & \text{for } r \leq 2^{1/6} \\ 0 & \text{for } r > 2^{1/6} \end{cases} \tag{S8}$$

$$U_{\mathrm{bond}}(r) = U_{\mathrm{WCA}}(r) + U_{\mathrm{FENE}}(r). \tag{S9}$$

In these expressions, r is once again the interparticle distance, K is a spring constant, and R_0 is the maximum bond length. In the Kremer-Grest model, $K = 30$, $R_0 = 1.5$, $\epsilon = 1$ and $\sigma = 1$ are used in the combined bond potential.

The 9-3 LJ potential we use to represent the substrate takes the form

$$U_{93}(r) = \epsilon_{93} \left(\frac{2}{15} \left(\frac{\sigma}{r} \right)^9 - \left(\frac{\sigma}{r} \right)^3 \right). \tag{S10}$$

This follows from the classical 12-6 LJ potential, integrated over a half-space of particles. It should be noted that ϵ_{93} does not directly equate to the ϵ of an individual interaction, although they are similar in magnitude.

Confirmation of degrafting

We checked for degrafting of the brushes using AFM measurements on separate brush samples. These samples were produced in the same batch as the ones used in the primary ellipsometry measurements, and left to degraft in the same vessel. Brush heights were checked prior to degrafting by scratching the surface with a steel needle, rinsing the surface with water and ethanol, then measuring the step height at the scratch. Corresponding height profiles and images are shown in figure S7.

After the degrafting period, one sample was again rinsed with water and ethanol, then measured. No scratch could be visually identified at this time, and surface features consisted mostly of irregular aggregates 20-30 nm in height. Height profiles and images are shown in figure S8. Since brushes are typically stable under normal rinsing and drying, we take this as indication that the degrafting procedure is succesful.

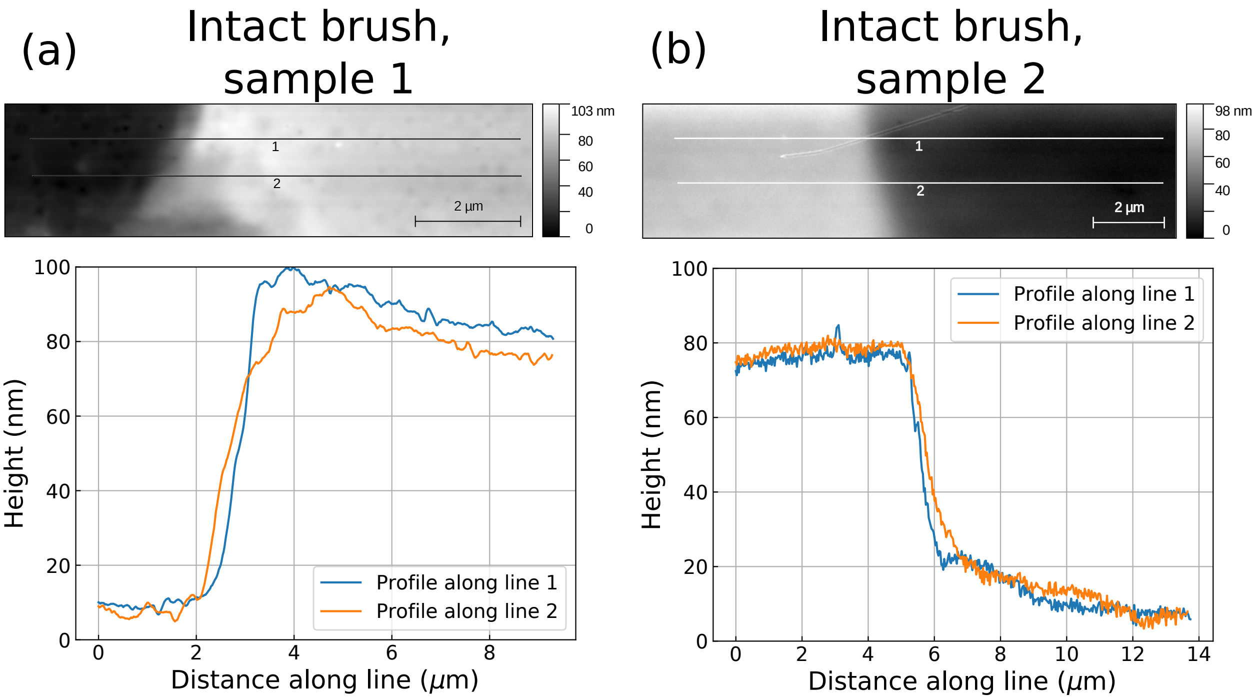

Figure S7: AFM height data and profiles across the edge of a scratch in the brush for two different brush samples.

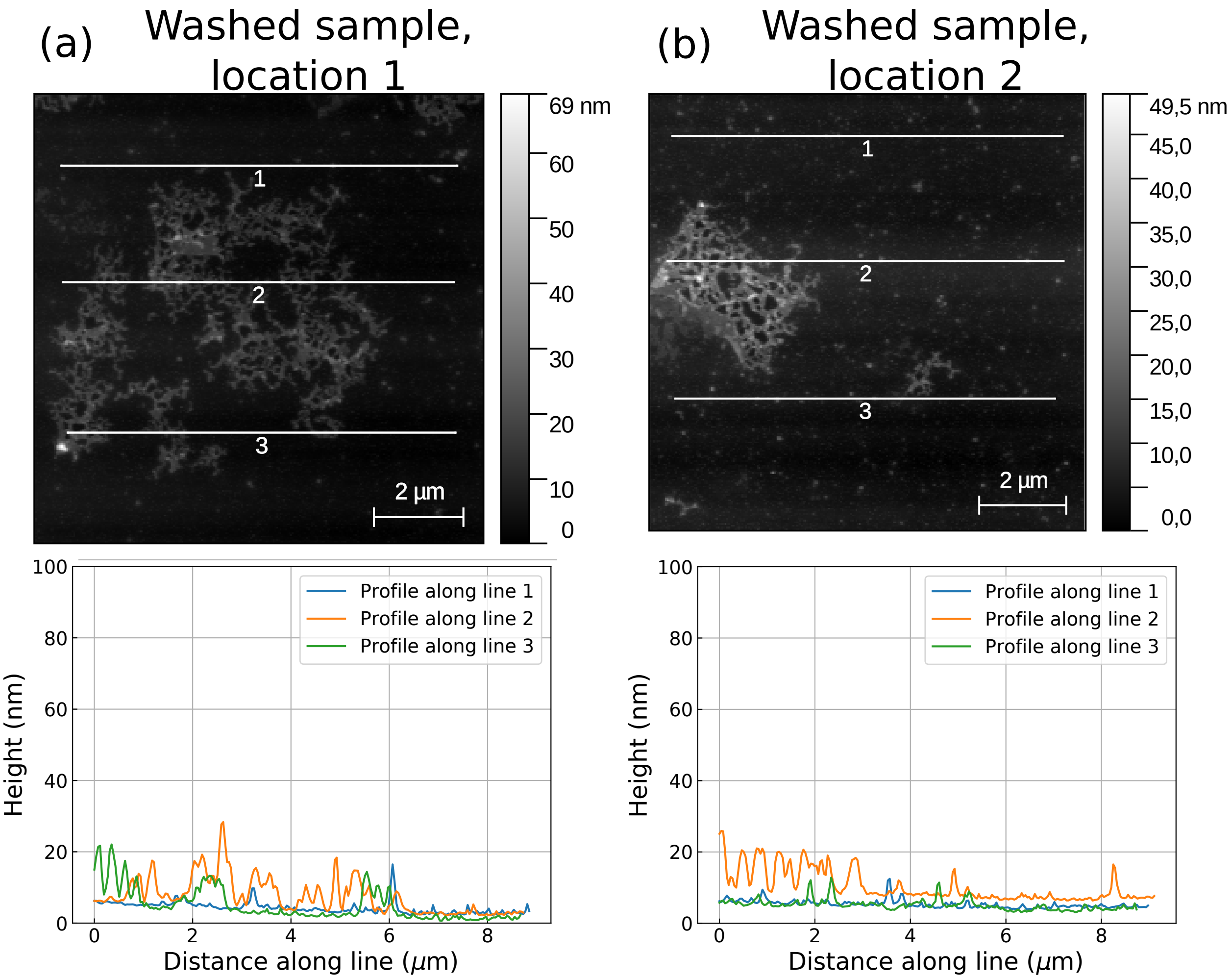

Figure S8: AFM height data and profiles of two spots on a brush sample after degrafting in saturated water vapor and rinsing.

Thickness non-uniformity

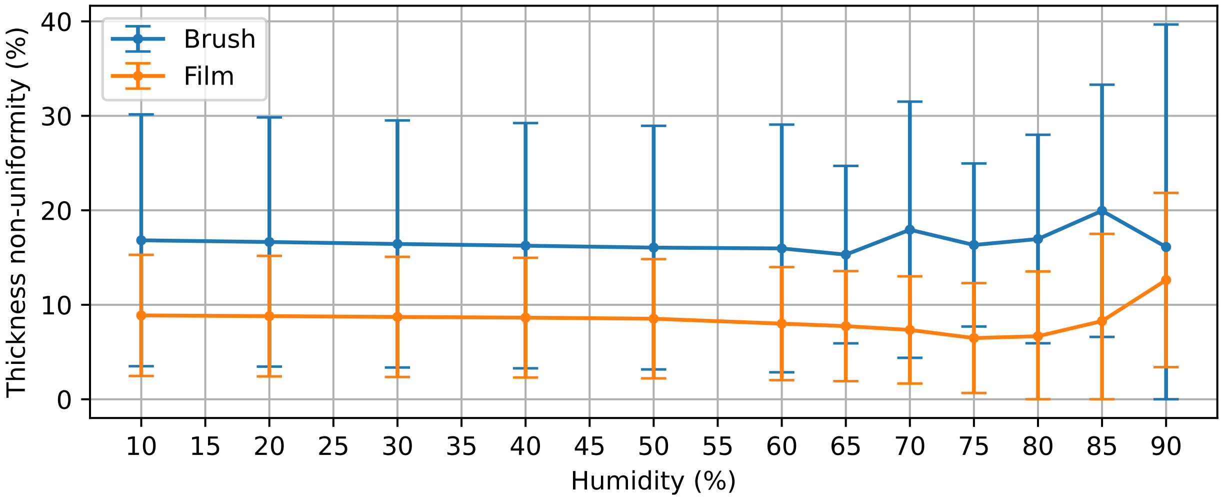

Figure S9: Average fitted thickness non-uniformity (with 95% confidence intervals) for brushes and films as a function of humidity.

References

(1) Kremer, K.; Grest, G. S. Dynamics of entangled linear polymer melts: A molecular-dynamics simulation. *The Journal of Chemical Physics* **1990**, *92*, 5057–5086.
